# *Bacillus clausii* Attenuates 5-Fluorouracil-Induced Intestinal Mucositis in Mice

**DOI:** 10.3390/ph17121676

**Published:** 2024-12-12

**Authors:** Francisco Ronaldo Farias Lima, Carlos Eduardo da Silva Monteiro, Samara Rodrigues Bonfim Damasceno, Patricia da Silva Pantoja, Álvaro Xavier Franco, Renan Oliveira Silva, Johnatan Alisson Oliveira Sousa, Tiago Santos Mendes, Marcos Aurélio Lima, Priscilla Fernanda Campos Justino, Marcellus Henrique Loiola Ponte de Souza, Pedro Marcos Gomes Soares

**Affiliations:** 1 Laboratory of Gastrointestinal Physio-Pharmacology (LEFFAG), Federal University of Ceará, Coronel Nunes de Melo Street, 1315 Rodolfo Teófilo, Fortaleza 60416-030, CE, Brazil; biofarias@hotmail.com (F.R.F.L.); kadusilvamonteiro@hotmail.com (C.E.d.S.M.); samararbdamasceno@hotmail.com (S.R.B.D.); patriciapantoj@gmail.com (P.d.S.P.); alv_17@yahoo.com.br (Á.X.F.); johnatanalisson@gmail.com (J.A.O.S.); tiagoedufisica@hotmail.com (T.S.M.); maslkim@hotmail.com (M.A.L.); prinand@gmail.com (P.F.C.J.); souzamar.ufc@gmail.com (M.H.L.P.d.S.); 2 Department of Physiology and Pharmacology, Federal University of Pernambuco, Av. da Engenharia-Cidade Universitária, Recife 50670-420, PE, Brazil; renan.oliveira25@yahoo.com.br; 3 Instituto Federal de Educação, Ciência e Tecnologia do Ceará-Campus Acopiara, Rodovia CE 060, Km 332, Vila Martins, Acopiara 63560-000, CE, Brazil; 4 Department of Morphology, Medical School, Federal University of Ceara, Rua Delmiro de Farias s/n, Rodolfo Teofilo, Fortaleza 60416-030, CE, Brazil

**Keywords:** probiotic, mucositis, 5-fluorouracil, *Bacillus clausii*, chemotherapy

## Abstract

5-Fluorouracil (5-FU) is an antimetabolite widely prescribed in cancer treatments, but its use in highly proliferative tissues can cause significant problems such as mucositis. *Bacillus clausii* is a probiotic commonly used for protection against acute diarrhea, gastrointestinal dysbiosis and inflammatory bowel diseases. We investigated the effect of *B. clausii* on 5-FU intestinal mucositis in mice. *B. clausii* was administered concomitantly with 5-FU on the first day and alone for the other two days. After the third day of 5-FU (450 mg/kg, ip), the animals were euthanized. Ileum samples were removed for evaluation for histopathological and biochemical analyses (myeloperoxidase (MPO), glutathione (GSH), malondialdehyde (MDA), catalase (CAT), interleukin-1 beta (IL-1β) and tumor necrosis factor alpha (TNF-α). In addition, we investigated gastric emptying and intestinal transit, intestinal permeability, intestinal smooth muscle contractility, transepithelial electrical resistance and intestinal transport of water and electrolytes. *B. clausii* reduced histopathological scores and increased the villus/crypt ratio in all intestinal segments after mucositis. *B. clausii* attenuated 5-FU-induced weight loss. The probiotic treatment increased GSH levels, reduced MPO and CAT activity, and also reduced MDA, IL-1β and TNF-α levels. *B. clausii* attenuated the delay in gastric emptying, water and electrolyte secretion and intestinal hypercontractility, and increased 5-FU-induced intestinal permeability. Thus, our data suggest that *B. clausii* may be a potential candidate for the treatment of chemotherapy-induced intestinal mucositis.

## 1. Introduction

Intestinal mucositis is one of the most common side effects related to chemotherapy with antimetabolites such as 5-FU [1]. 5-FU is widely used in gastrointestinal, pancreatic, breast and head/neck cancers, alone or in combination with other drugs or radiotherapy. Clinical symptoms of 5-FU-induced intestinal mucositis include nausea, vomiting, dyspepsia, dysphasia and diarrhea [2].

In experimental models, 5-FU provoked important histopathological and inflammatory changes in the small intestine [3,4,5]. It is also associated with a delay in gastrointestinal motility and hypercontractility in the gastric fundus and duodenum muscle [3].

Probiotics may restore gut microbiome composition and introduce beneficial functions to gut microbial communities, consequently ameliorating or preventing gut inflammation and other intestinal diseases [6]. *B. clausii* is an important probiotic, used orally in prophylaxis against pathogenic bacteria and diarrhea. *B. clausii* in spore form is heat-stable, can be stored at room temperature, has an advantage over other non-spore formers such as *Lactobacillus spp.* and is capable of surviving the low pH of the gastric barrier [7,8,9] which is not the case for all species of *Lactobacillus* [10].

*B. clausii* spores have been used in the treatment or prevention of gut barrier impairment, and their effects have been related to several properties, such as antimicrobial and immunomodulatory activity [7,11]. In this context, we investigated whether treatment with *B. clausii* is effective against the physiological, biochemical and morphological changes caused by 5-FU-induced intestinal mucositis in mice.

## 2. Results

### 2.1. Effect of B. clausii on Weight Loss and Leukopenia Induced by 5-FU

**Figure 1:**
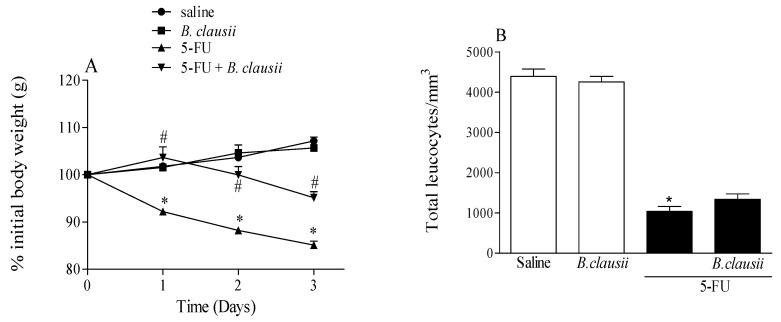
Effect of *B. clausii* treatment on weight loss (**A**) and leukopenia (**B**) induced by 5-FU in mice. The *B. clausii* probiotic was administered for three days and weighed daily throughout the experiment. Values are represented as means ± SEM (*n* = 6). * *p* < 0.05 vs. control and ^#^ *p* < 0.05 vs. 5-FU. ANOVA followed by Bonferroni’s test was used for statistical analyses.

In Figure 1A, 5-FU caused a significant body weight loss demonstrated on the last day (72.57 ± 1.4% 5-FU group vs. control: 107.2 ± 0.84%). *B. clausii* attenuated the weight loss. Furthermore, *B. clausii* alone did not alter the body weight.

5-FU induced leukopenia (1038 ± 123.1 total leucocytes/mm^3^ vs. saline 4394 ± 182.6 total leucocytes/mm^3^). The 5-FU-Bc group did not change the leukopenia compared to the 5-FU group (1339 ± 133.3 total leucocytes/mm^3^ vs. 5-FU 1038 ± 123.1 total leucocytes/mm^3^) (Figure 1B).

### 2.2. B. clausii Reduce Histopathological and Morphometry Changes

**Figure 2:**
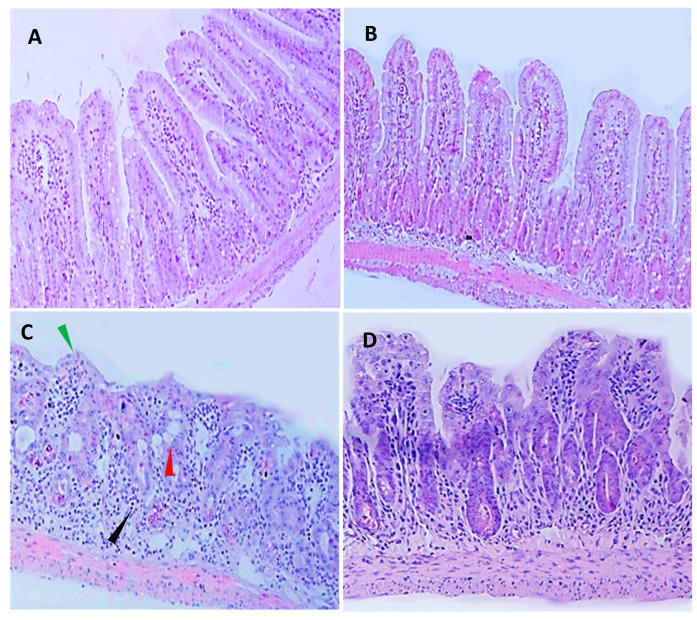
Effect of *B. clausii* on histopathological changes in the ileum of mice with intestinal mucositis induced by 5-FU. Photomicrographs (100×) showing the ileal segment of the control group (saline, **A**), *B. clausii* + saline (**B**), 5-FU (**C**) and 5-FU+ *B. clausii* (**D**). Black arrows indicate the presence of neutrophil infiltrate, a red arrowhead indicates the presence of vacuolated cells and a green arrowhead indicates a shortened villous.

5-FU caused histological alterations in the ileum (vacuolated and shortened villous) and intense inflammatory infiltrates (Figure 2C). The 5-FU-Bc group had a significant reduction in histopathological alterations on the ileum (Figure 2D, Table 1).

**Figure 3:**
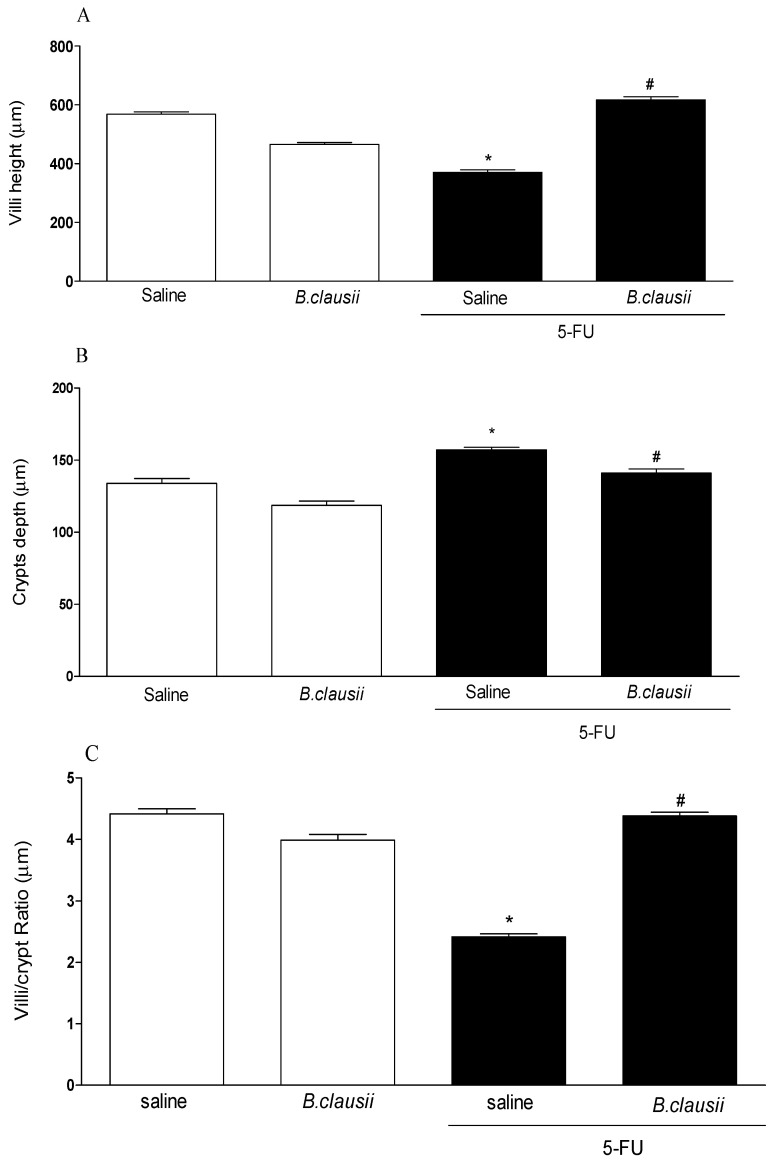
*B. clausii* reduces the morphometry changes caused by 5-FU administration. The villus height (panel **A**), crypt depth (panel **B**) and villus/crypt ratio (panel **C**) were measured on the ileal segment. The values are represented as means ± SEM (*n* = 6). * *p* < 0.05 vs. control and ^#^ *p* < 0.05 vs. 5-FU. ANOVA followed by Bonferroni’s test was used for statistical analyses.

The 5-FU group had a significant reduction in the villus height (Figure 3A), an increase in the crypt depth (Figure 3B) and a reduction in the villus/crypt ratio (Figure 3C) of the ileum segment compared to the saline group. The 5-FU-Bc group had significant attenuation of the altered parameters (Figure 3A–C).

### 2.3. B. clausii Effects on MPO and Catalase Activity, Glutathione, Malonaldehyde and Cytokine Levels

**Figure 4:**
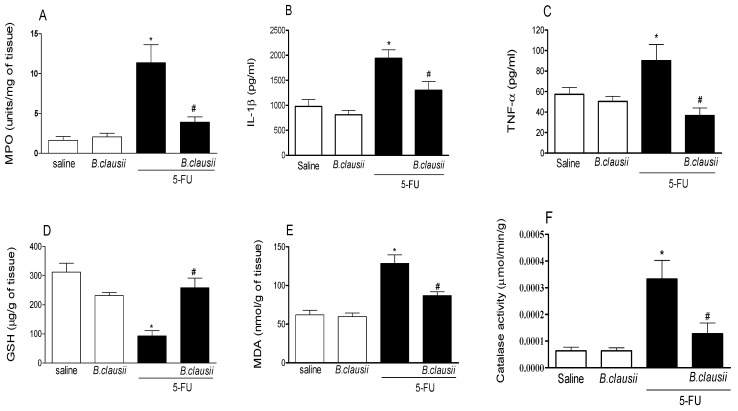
*B. clausii* effects on MPO activity (**A**), IL-1β (**B**) and TNF-α (**C**) levels, glutathione (**D**), malonaldehyde (**E**) and catalase (**F**) levels in the ileum of mice with 5-FU induced intestinal mucositis. Values are represented as means ± SEM (*n* = 6). * *p* < 0.05 vs. control and ^#^ *p* < 0.05 vs. 5-FU. ANOVA followed by Bonferroni’s test was used for statistical analyses.

Figure 4 shows that 5-FU resulted in a significant increase in MPO activity (85.8%) when compared to the saline group. *B. clausii* resulted in lower MPO activity (65.5%) compared to the 5-FU group (Figure 4A).

Pro-inflammatory cytokine levels were significantly increased in the 5-FU group (TNF-α: 36.9%; IL-1β: 49.5%) compared to the saline group. However, treatment with *B. clausii* (TNF: 59.3% and IL-1β: 32.8%) significantly reduced these levels (Figure 4B,C).

GSH concentrations in the 5-FU group (70.2%) were significantly lower, when compared to the saline group. *B. clausii* treatment (64%) restored GSH content to saline levels (Figure 4D).

MDA concentrations in the saline group increased after 5-FU administration (51.7%). *B. clausii* significantly reduced MDA concentrations (32.2%) compared to the 5-FU group (Figure 4E).

Figure 4F revealed the increased catalase activity in the 5-FU group (81.8%) compared to the saline group. After treatment with *B. clausii*, catalase activity was significantly reduced in the 5-FU group (60.6%).

### 2.4. B. clausii Ameliorate the Gastric Emptying and Intestinal Transit

**Figure 5:**
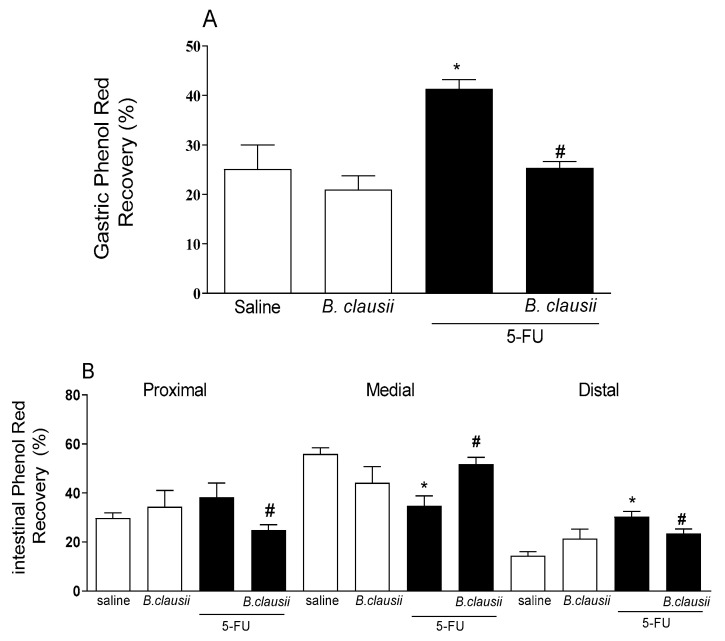
*B. clausii* effects on delayed gastric emptying (panel **A**) and intestinal transit (panel **B**) associated with 5-FU-induced intestinal mucositis in mice. Values are represented as means ± SEM (*n* = 6). * *p* < 0.05 vs. control and ^#^ *p* < 0.05 vs. 5-FU. ANOVA followed by Bonferroni’s test was used for statistical analyses.

Figure 5A demonstrates that gastric retention was significantly higher in the 5-FU group (39.2%) when compared to the saline group. *B. clausii* displayed a significant decrease (38.7%) compared to the 5-FU group.

Gastrointestinal transit was significantly slower in the 5-FU group than in the saline group. *B. clausii* treatment attenuated retention on proximal and medial intestinal segments compared to the 5-FU group. However, this effect was not observed in the distal region (Figure 5B).

### 2.5. B. clausii Restores the Intestinal Transport of Water and Electrolytes

**Figure 6:**
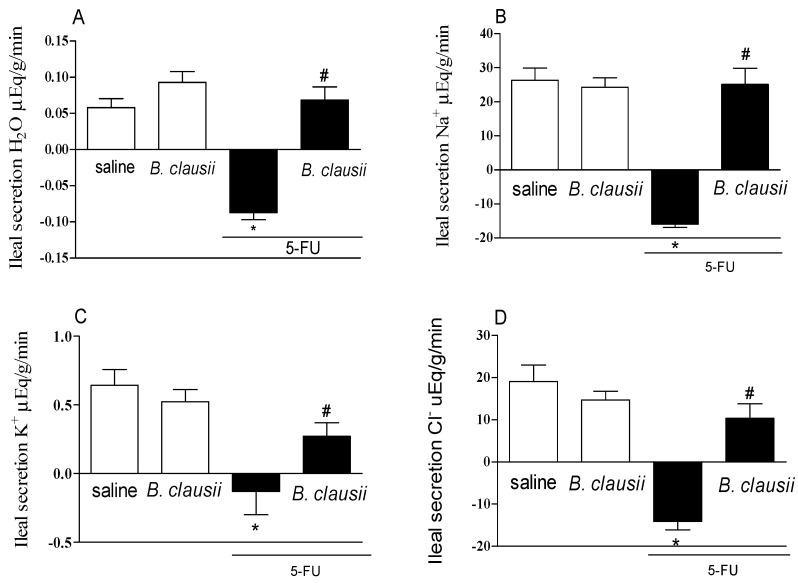
*B. clausii* restores the intestinal transport of water (panel **A**) and electrolytes (Na^+^/panel **B**; K^+^/panel **C** and Cl^−^/panel **D**) in mice with 5-FU-induced intestinal mucositis. Values are represented as means ± SEM (*n* = 6). * *p* < 0.05 vs. control and # *p* < 0.05 vs. 5-FU. ANOVA followed by Bonferroni’s test was used for statistical analyses.

Figure 6A–D, 5-FU significantly increased the secretion of H_2_O, Na^+^, K^+^ and Cl^−^ (H_2_O: −0.087 ± 0.009 mL/g/min; Na^+^: −15.98 ± 0.927 μEq/g/min; K^+^: −0.130 ± 0.169 μEq/g/min; Cl^−^: −14.12 ± 2.029 μEq/g/min) compared to the saline group (H_2_O: 0.057 ± 0.012 mL/g/min; Na^+^: 26.34 ± 3.588 μEq/g/min; K^+^: 0.642 ± 0.115 μEq/g/min; Cl^−^: 19.06 ± 3.932 μEq/g/min). *B. clausii* restored the transport of water and electrolytes compared to the 5-FU group (Figure 6A–D).

### 2.6. Effect of B. clausii on Contractile Response in Ileal Smooth Muscle

**Figure 7:**
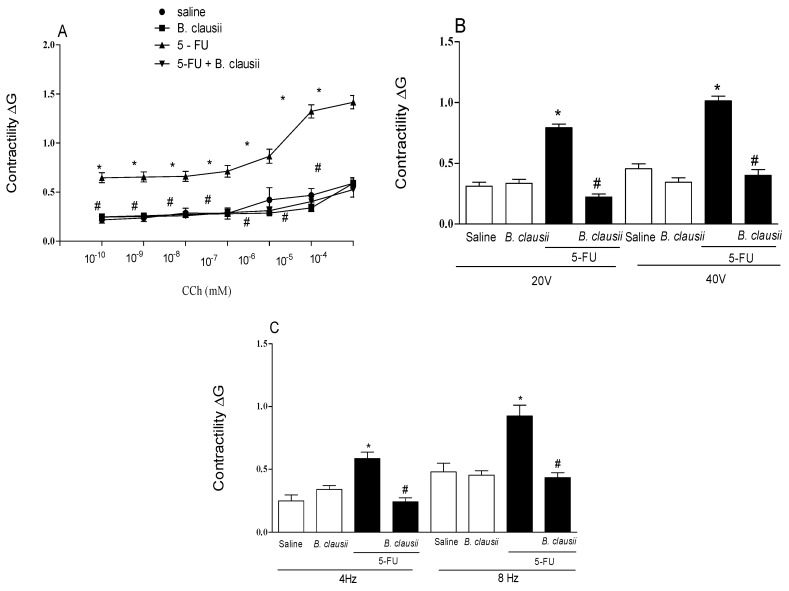
Effect of *B. clausii* on contractile response in ileal smooth muscle stimulated by carbachol (panel **A**), voltage (panel **B**) and frequency curve of electrical stimulation (panel **C**) in mice with 5-FU-induced intestinal mucositis. Values are represented as means ± SEM (*n* = 6). * *p* < 0.05 vs. control and ^#^ *p* < 0.05 vs. 5-FU. ANOVA followed by Bonferroni’s test was used for statistical analyses.

Figure 7A–C, 5-FU induced alteration of mechanical activity of gastrointestinal smooth muscle, resulting in hypercontractility of the ileal smooth muscle (57.1%) when stimulated by carbachol in cumulative concentrations (10^−10^–10^−4^ mM) (Figure 7A). *B. clausii* reversed the hypercontractility on the ileum (64.3%) when compared to the 5-FU group.

The contractile response of the small intestine to an electrical stimulation field was altered by 5-FU, resulting in hypercontractility of the ileal smooth muscle to voltage stimulus compared to the saline group (Figure 7B). The volt stimulus responses of 20 V (62.5%) and 40 V (60%) were higher in the 5-FU group than those in the saline group. The 5-FU-Bc group had a reduction in contractions post electrical stimulation.

Contractility in response to frequency stimulation (4 Hz) increased on the ileum of 5-FU animals (66.7%) when compared to the saline group. The 5-FU-Bc group had a reduction (66.7%) of the contractile response compared to the 5-FU group. The frequency of 8 Hz increased contractile response on the ileum (44.4%) in the 5-FU group. The treatment with *B. clausii* reduced the contractile response (55.5%) compared to the 5-FU group (Figure 7C).

### 2.7. B. clausii Effects on Intestinal Permeability and Transepithelial Electrical Resistance

**Figure 8:**
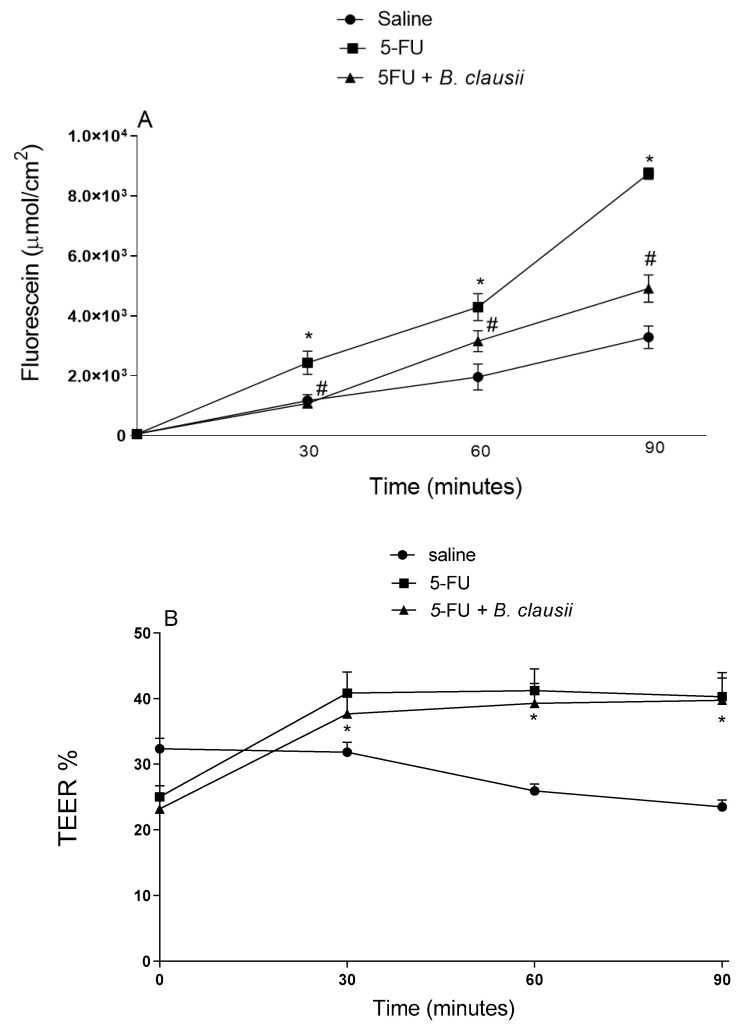
*B. clausii* effects on intestinal permeability (panel **A**) and transepithelial electrical resistance intestinal/TEER (panel **B**) in mice with 5-FU-induced intestinal mucositis. Values are represented as means ± SEM (*n* = 6). * *p* < 0.05 vs. control and ^#^ *p* < 0.05 vs. 5-FU. ANOVA followed by Bonferroni’s test was used for statistical analyses.

Figure 8A shows that 5-FU significantly increases the ileal mucosal permeability to fluorescein at 90 min (58.3%) when compared to the saline group. *B. clausii* significantly decreased the passage of fluorescein, as indicated in Figure 8A.

5-FU induced a significant increase in ileal transepithelial electrical resistance (TEER) at 90 min compared to the saline group (41.6%). *B. clausii* does not reduce the ileal transepithelial electrical resistance (Figure 8B).

## 3. Discussion

Our group has demonstrated that probiotics were able to reduce the alterations of the functional events and inflammatory process during 5-FU-induced mucositis in mice [4,5,12] and cells [13] by reducing several inflammatory parameters that included the production of pro-inflammatory cytokines as well as oxidative stress and restoring intestinal motility [4,5,12]. Intestinal mucositis is known to potentially influence the local microbiota, just as intestinal dysbiosis is involved in inflammatory changes, permeability, tissue repair and the release of immune system factors, such as cytokines, present in chemotherapy toxicity [14].

We found that *B. clausii* promotes mucosal protection by attenuating damage in the 5-FU-induced epithelial barrier function and enhancing histopathological and biochemical alterations. *B. clausii* is an aerobic, spore-forming bacterium that is able to inhibit the growth of pathogens in the gastrointestinal tract via three distinct mechanisms: colonization of free ecological niches; competition for epithelial cell adhesion, which is particularly relevant for spores in the initial or intermediate germination phase; and the production of antibiotics and/or enzymes secreted into the intestinal environment, especially peptide antibiotics [15]. These spore-forming bacteria are extremely stable and resistant, can survive the low pH of the gastric barrier and reach the intestine intact [16]. In addition, spore-based products can be indefinitely stored without refrigeration or in a desiccated form without any deleterious effect on viability [17].

*B. clausii* spores have been used in the treatment or prevention of gut barrier impairment, and their effects have been related to several properties, such as antimicrobial and immunomodulatory activity [7,11], regulation of cell growth and differentiation, cell–cell signaling, cell adhesion, signal transcription and transduction [18], production of vitamins [19] and gut protection from genotoxic agents [20].

*B. clausii* seems not to affect 5-FU-dependent leukopenia, which represents one of the routes of toxicity of its antineoplastic potential. 5-FU exerts its therapeutic effects by causing cytotoxicity in cancer cells, thereby preventing cell proliferation and inducing apoptosis [21]. However, due to its non-specificity, 5-FU can also cause damage to normal cells. Particularly, 5-FU affects tissues characterized by rapid cell proliferation, including the bone marrow, thereby promoting extensive leukopenia in patients undergoing treatment [22,23].

The use of antineoplastic drugs causes a loss of body weight. This important symptom is associated with 5-FU-induced intestinal mucositis [6] caused by reduced food absorption due to alterations in the intestinal villi and loss of water and electrolytes caused by diarrhea [24]. *B. clausii* probiotics have been described as an antidiarrheal that prevents antibiotic-associated diarrhea [25]. Data describing the functional mechanisms remain very limited. 5-FU reduction in body weight in mice was prevented by *B. clausii*; these data are consistent with research by our group using others probiotics (*Lactobacillus spp.* and *Bifidobacterium spp.*) in intestinal mucositis caused by 5-FU [5,12].

*B. clausii* improved histopathological parameters, loss of mucosal architecture of the duodenum, jejunum and ileum, inflammation scores, the architecture and the total thickness of the intestinal mucosa that were altered in mucositis caused by 5-FU. This improvement occurs due to the reduction in inflammatory and oxidative markers corroborating other studies that have shown that treatment with probiotics attenuates inflammation in 5-FU-induced mucositis in mice, reducing inflammatory parameters and markers of oxidative stress and restoring intestinal motility [13,26].

Probiotics have beneficial effects that are also attributed to their ability to modulate the immune response [27]. The epithelial damage developed in intestinal mucositis caused by 5-FU presents extensive migration of neutrophils associated with oxidative stress, where there is an increase in myeloperoxidase activity under neutrophil infiltration, directly associated with the inflammatory condition in the intestine. In this study, we demonstrate that *B. clausii* treatment reduces myeloperoxidase activity, lipid peroxidation and the consumption of glutathione (GSH), a multifunctional intracellular antioxidant, which enhances a crucial role in the elimination of free substances in the gastrointestinal tract [28], which are increased with the use of 5-FU corroborating previous studies that have shown that the administration of 5-FU alters the antioxidant defense system [3,5,12].

During intestinal mucositis, there is an increase in the expression of pro-inflammatory cytokines, and this increase depends on the activation of the Toll-like/MyD88/NF-kB/MAPK pathway, as demonstrated by Justino et al. [13]. Thus, it is known that there is an activation of immune activity during intestinal mucositis. Other studies have demonstrated the immunomodulatory activity of *B. clausii*, reducing the expressions of IL-4, IFN-γ, IL-12, TGF-β and IL-10 in allergic children with recurrent respiratory infections [29]. Marseglia et al. [15] showed that the immune responses of *B. clausii* include the relief of nasal symptoms during allergic reactions and the anti-inflammatory effect against the side effects of Helicobacter pylori antibiotic therapy.

Another study by Paparo et al. [30] demonstrated that probiotic strains of *B. clausii* and their metabolites had beneficial effects on Caco2 cells by increasing the synthesis of the antimicrobial peptides of the innate immunity HBD-2 and LL-37, which are responsible for effective defense mechanisms against various pathogens in the gastrointestinal tract, and also inhibited the production of ROS and the release of pro-inflammatory cytokines (IL-8 and IFN-β) in Rotavirus-infected cells.

Regarding intestinal functional alterations, it is known that the ileal segment has been studied with greater functional repercussions than the other segments. Therefore, our functional analysis was limited to the ileal segment. 5-FU promotes oxidative and inflammatory processes, in addition to inducing changes in gastrointestinal motility. Some authors attribute the delay in gastric emptying and intestinal transit to the changes in intestinal motility imposed by the intestinal inflammatory process [3,4]. Our findings showed that *B. clausii* attenuates 5-FU-induced gastrointestinal dysmotility, increasing intestinal transit and gastric emptying, according to Soares et al. [3] and Justino et al. [4].

Rtibi et al. [31] demonstrated that the chemotherapeutic agent 5-FU induces alterations in the transport of mucosal fluids and electrolytes, resulting in a hypersecretory response and increased defecation. In this context, we showed that 5-FU administration altered fluid and electrolyte concentrations in the small bowel. Both actions were accompanied by changes in water and electrolyte transport across the intestinal mucosal membrane.

Our findings demonstrate that *B. clausii* prevented functional alteration assessed in ileal smooth muscle hyper-contractility caused by 5-FU in the inflammatory phase. Soares et al. [3] observed duodenal hyper-contractility after systemic 5-FU administration in rats. This hyper-contractility was reported in the inflammatory (third day after 5-FU administration) and post-inflammatory (15th day after 5-FU administration) phases.

Given that neutrophil migration across mucosal epithelia is associated with increased epithelial permeability and disruption of critical barrier function in diseases such as inflammatory bowel disease [32], intestinal mucositis encompasses an increase in intestinal permeability and villus and crypt atrophy, thus leading to severe loss of function of the epithelial barrier [26]. In this present study, *B. clausii* reduced intestinal permeability by reducing the inflammation associated with intestinal mucositis. Supporting our findings, Justino et al. [4] demonstrated that mice with 5-FU-induced intestinal mucositis had a significant change in mucosal integrity and that treatment with *S. boulardii* improves intestinal permeability in these animals. Paparo et al. [30] demonstrated that probiotic strains of *B. clausii* and their metabolites had beneficial effects on markers of epithelial barrier (mucin protein MUC5AC and occluding and ZO-1 tight junction proteins) damage and enterocyte monolayer permeability in Rotavirus-infected cells. This study demonstrated that *B. clausii* is capable of positively reversing an alteration in the integrity of the intestinal mucosa.

## 4. Materials and Methods

### 4.1. Evaluation of Weight Loss and Blood Leukocyte Counts

We evaluated the weight loss of the animals during the three days after 5-FU administration [3]. Blood samples obtained by cardiac puncture of male Swiss mice were transferred to test tubes, heparinized and diluted in Turk’s solution (380 µL of blood and 20 µL of diluent solution). Then, the cells were counted in a Neubauer chamber under a light microscope [4].

### 4.2. Animals

Male Swiss mice (25–30 g) were obtained from the Department of Physiology and Pharmacology, Federal University of Ceará, Brazil. Mice were housed under a 12 h light/dark cycle in a temperature-controlled room with ad libitum access to water. Mice were fasted for 24 h with standard chow pellets (Nuvilab Chow, Nuvital, Colombo, PR, Brazil). Animal studies were approved by the Institutional Animal Care and Use Committee (protocol #141/2014). All procedures involving animals were performed in accordance with the Guide for Care and Use of Laboratory Animals of the US Department of Health and Human Services.

### 4.3. 5-Fluorouracil-Induced Intestinal Mucositis Model

Spores from four strains of *B. clausii* were used: O/C, N/R, T, and SIN. As strains are so designated due to their antimicrobial resistance profiles, they are O/C resistant to chloramphenicol, N/R resistant to novobiocin and rifampicin, T resistant to tetracycline and SIN resistant to neomycin and streptomycin [33].

Swiss mice (male) were randomly divided into a saline group (*n* = 6), *B. clausii* group (saline + *B. clausii* 1.0 × 10^9^ CFU/ 2.5 mL in 3 days, *n* = 8), 5-FU group (saline + 5-FU 450 mg/kg, single dose, i.p. *n* = 6) and a 5-FU + *B. clausii* group (5-FU 450 mg/kg, single dose, i.p. + *B. clausii* 1 × 10^9^ CFU/ 2.5 mL in 3 days, *n* = 6). The mice were euthanized three days after 5-FU administration. Blood samples were taken for a leukocyte count. Intestinal samples (ileum segment) were removed for analysis of morphological and histopathological features, myeloperoxidase activity, malondialdehyde, catalase, interleukin-1 beta and tumor necrosis factor alpha cytokines. Analyses of gastric emptying, gastrointestinal transit, intestinal transportation of water and electrolysts, contractility of smooth muscle, intestinal permeability and transepithelial electrical resistance were also performed. The animals were weighed daily throughout the experiment.

### 4.4. Intestinal Morphometry and Histopathology

Ileum segments were collected, fixed and stained with hematoxylin and eosin for the measurement of villus height and crypt depth. Ten intact and well-oriented villi and crypts were measured for each sample, chosen randomly. The microscopy analysis was double-blinded using an optical microscope with a millimeter eyepiece or image acquisition system and measurement software AVIVA 14.0 (LEICA, Washington, DC, USA). Histopathological scores used were according with modification to MacPherson and Pfeiffer [34]. Score 0: normal histological findings; Score 1: Mucosa: villus blunting, loss of crypt architecture, sparse inflammatory cell infiltration, vacuolization and edema. Normal muscular layer; Score 2: Mucosa: villus blunting with fattened and vacuolated cells, crypt necrosis, intense inflammatory cell infiltration, vacuolization and edema. Normal muscular layer; Score 3: Mucosa: villus blunting with fattened and vacuolated cells, crypt necrosis, intense inflammatory cell infiltration, vacuolization and edema.

### 4.5. MPO Activity and Cytokine Levels

MPO activity was evaluated using colorimetric analysis of the intestinal segments according to the method described by Bradley et al. [35]. Briefly, intestinal tissue (50 mg/mL) was homogenized in HTAB buffer (Sigma-Aldrich, St. Louis, MO, USA). The homogenate was centrifuged at 1500× *g* for 15 min at 4 °C. MPO activity in the supernatants was evaluated using o-dianisidine dihydrochloride (Sigma-Aldrich, St. Louis, MO, USA) and 1% hydrogen peroxide (Merck, Whitehouse Station, NJ, USA) with detection at 450 nm. The results were expressed as MPO units/mg tissue.

For cytokine levels, ileal segments (100 mg of tissue) were homogenized in 1 mL of 1× PBS and centrifuged at 1500× *g* for 10 min. TNF-α and IL-1β concentrations were measured using ELISA DuoSet Kits (R&D Systems, Minneapolis, MN, USA, DY401 and DY410) at 450 nm. Cytokine concentrations are expressed as picograms per milliliter (pg/mL) and were measured using a method described previously by da Silva Monteiro et al. [36].

### 4.6. Glutathione and Malondialdehyde Content and Catalase Activity

The ileum segment was homogenized in 0.02 M EDTA (1 mL/100 mg of tissue). Homogenate (400 μL) was mixed with 320 μL of distilled water and 80 μL of 50% trichloroacetic acid (TCA) to precipitate proteins. The samples were centrifuged at 3000× *g* for 15 min at 4 °C. Thus, 400 μL of supernatant were mixed with 800 μL of 0.4 M Tris buffer (pH 8.9) and 20 μL of 5.5-dithiobis-(2-nitrobenzoic acid) (DTNB) (Fluka, St. Louis, MO, USA), followed by shaking for 3 min. Absorbance was measured at 412 nm. The results were expressed as μg GSH/mg tissue [37].

For MDA, samples (100 mg of tissue) were homogenized in 1.15% KCl (1 mL), followed by an addition of 1% H3PO4 and 0.6% tert-butyl alcohol. The mixture was stirred and heated in a boiling water bath for 45 min and cooled immediately in an ice-water bath with the addition of n-butanol (2 mL). The mixture was stirred and centrifuged at 1500× *g* for 10 min, and absorbance was measured at 520 and 535 nm. Results were expressed as nmol/mL of homogenate [38].

Catalase activity was measured according to the transformation from hydrogen peroxide to H_2_O and O_2_. An aliquot (0.1 mL) of supernatant was added to 1.5 mL of phosphate buffer and 1 mL of distilled water. After preheating at 25 °C, 0.3 mL of H_2_O_2_ was added to the reaction solution. Absorbances were recorded at 240 nm for 4 min. The results are expressed as mM/min/μg protein [39].

### 4.7. Functional Evaluation of Intestine Segments

#### 4.7.1. Gastric Retention and Gastrointestinal Transit

Gastric emptying was evaluated using a modification of the methodology described by Reynell and Spray [40]. Mice received the last treatment 30 min prior to the gastric emptying experiment. The mice were fed a standard liquid bolus (300 μL) containing a nonabsorbable marker (0.75 mg/mL phenol red in 5% glucose) by gavage. The animals were euthanized, followed by laparotomy to expose the stomach and the bowels. The esophageal-gastric, gastroduodenal and ileocecal junctions were immediately isolated using ligatures. The segments were removed and divided into the stomach and proximal, medial and distal bowels. The samples were cut into small pieces and homogenized (solution of 100 mL 0.1 N NaOH). One milliliter of supernatant was collected and centrifuged for 10 min at 2000× *g*.

Proteins of the homogenates were precipitated by adding 20% TCA, and the samples were centrifuged for 20 min at 2000× *g*. 150 mL of supernatant was collected and added to 200 mL of 0.5 N NaOH. The absorbance was measured at 540 nm using a spectrophotometer. The fractional dye retention was expressed as a percentage according to the following equation: gastric dye retention = amount of phenol red recovered in stomach/total amount of phenol red recovered from two segments (stomach and small intestine).

Intestinal transit was calculated for each bowel segment, dividing the amount of phenol red recovered from a given segment by the amount of phenol red recovered from all three segments and expressed as a percentage.

#### 4.7.2. Intestinal Fluid and Electrolyte Transport

The effects of *B. clausii* on ileal fluid and electrolyte transport were assessed on the last day of the experimental protocol. Mice were divided into 4 groups (Saline, *B. clausii*, 5-FU and 5-FU + *B. clausii* groups, *n* = 6) and submitted to intestinal perfusion to evaluate net fluid and electrolyte transport.

Mice were fasted for 18 h ad libitum before each experiment. The animals were anesthetized with ketamine (35 mg/kg, i.m.) and xylazine (5 mg/kg, i.m.) and performed a median 3 to 5 cm laparotomy for visualization of the small intestine. After the surgery, the infusion was performed under a peristaltic pump (Control Company, Edgewood Friendswood, TX, USA) of Tyrode’s solution without glucose (NaCl 8.0 g/L, KCl 0.35 g/L, MgCl_2_ 0.1 g/L, CaCl_2_ 0.2 g/L, NaHCO_3_ 1 g/L, NaH_2_PO_4_ 0.05 g/L and phenol red 0.05 mg/mL as a nonabsorbable label) at 0.14 mL/min for 60 min. The perfusate was collected in tubes every 20 min for 60 min of total infusion (T20, T40 and T60 samples). These tubes were then heated in a water bath at 37 °C. Differences between values of Na^+^, K^+^ and Cl^−^ concentrations were used to calculate the rate of ileal transport of electrolytes. Differences between phenol red concentrations were used to evaluate the secretion/absorption rates of water by perfused tissues. The secretion and absorption parameters of electrolytes and water were corrected by the time and mass of the perfused ileal segment, as described by Lima et al. [41].

#### 4.7.3. Contractility Measures

Longitudinal strips (1 cm) of the ileum of the groups were removed and washed with Krebs solution (118.3 NaCl, 4.7 KCl, 2.5 CaCl_2_, 1.2 MgSO_4_, 1.2 KH_2_PO_4_, NaHCO_3_ 25.0 and 11.1 C_6_H_12_O_6_ mmol/L, pH = 7.4). Subsequently, strips were mounted in organ bath chambers with 5 mL of Krebs solution at 37 °C, pH = 7.4, bubbled with 5% CO_2_ and 95% O_2_ [42]. One end is tied to a lever and the other to an isometric tension transducer to measure contraction force (model Panlab S.L., Barcelona, Spain). The tissues were equilibrated under a tension of 1 g for 60 min, during which 0.1 nM–1 mM carbachol and 60 mM KCl were applied repeatedly until the reactivity of the tissues became reproducible.

For the experiments of electric stimulation, we used the following electrical parameters: voltages over a range of 20 and 40 V, 1 ms and a duration of 10 s. A 4 to 8 Hz frequency curve of the stimulated tissues was then obtained in comparison with the contractions induced by 60 mM KCl.

#### 4.7.4. Intestinal Permeability and Transepithelial Electrical Resistance (TEER)

Ileal mucosal segments were mounted in a diffusion chamber to measure permeability through fluorescein dye (376 Da; 1 mg/mL, diluted in KHBB, pH 7.4) [43]. After a stabilization period with KHBB (pH 7.4, 30 min), the solutions on the luminal side were replaced by solutions containing the fluorescent marker. The luminal contents (100 µL) were collected from the serosal side in 30 min intervals (0, 30, 60, 90 and 120) for 2 h. The fluorescent marker was measured using a fluorescence plate reader (FLUOstar Omega; BMG Labtech, Ortenberg, Germany).

The ileal segment was mounted in Ussing chambers (Mussler Scientific Instruments, Aachen, Germany) with a seromuscular layer and an exposed area of 0.096 cm^2^. The mucosa and serous layers were filled with Krebs-Ringer bicarbonate buffer, supplemented with 10 mM mannitol and 10 mM glucose, respectively. The solutions were maintained at 37 °C and gassed with carbogen. The difference in transmucosa potential was continuously monitored using Ag/AgCl electrodes. Transient electrical resistance (TEER) was calculated according to Ohm’s law from the voltage deflections induced by bipolar current pulses of 50 mA every 60 s with a duration of 200 ms. TEER values were recorded for each tissue at 30 min intervals. The mean TEER between 90 and 120 min was calculated for each experimental group. These times were selected because a stable TEER plateau was reached in most tissues between 90 and 120 min after assembly [44].

### 4.8. Statistical Analysis

Results were expressed as means ± standard error of the mean (SEM) for each group. Statistical analysis between groups was performed using the analysis of variance (ANOVA), followed by the multiple comparison test of Bonferroni and, for non-parametric data, Kruskal–Wallis and Dunn’s tests were used. Statistical significance was considered when *p* < 0.05. For statistical tests, we used GraphPad PRISM^®^ Software, version 5.

## 5. Conclusions

*B. clausii* is shown to ameliorate inflammatory and oxidative stress and attenuate gastrointestinal motility caused by the toxicity of 5-Fluorouracil. The findings contribute to the discovery of a new therapy for gastrointestinal toxicity caused by chemotherapy agents.

## Figures and Tables

**Table 1:** Histopathological parameters by *B. clausii* in mice with 5-fluorouracil (5-fu)-induced intestinal mucositis (median scores and ranges).

Groups *n* = 8	Ileum
Saline	0(0–2)
*B. clausii*	1(0–2)
5-FU	3(2–3) *
5-FU + *B. claussi*	2(1–3) ^#^

* *p* < 0.05 vs. saline and ^#^ *p* < 0.05 vs. 5-FU.

## Data Availability

Data are contained within the article.
